# TLR2 and TLR9 modulate enteric nervous system inflammatory responses to lipopolysaccharide

**DOI:** 10.1186/s12974-016-0653-0

**Published:** 2016-08-18

**Authors:** Joan F. Burgueño, Albert Barba, Elena Eyre, Carolina Romero, Michel Neunlist, Ester Fernández

**Affiliations:** 1 Department of Cell Biology, Physiology and Immunology, Universitat Autònoma de Barcelona, Bellaterra, Barcelona, 08193 Spain; 2 INSERM, UMR913, Nantes, F-44093 France

**Keywords:** Enteric nervous system, Enteric neuron, TLR2, TLR4, TLR9, Inflammation, Chemoattraction

## Abstract

**Background:**

Accumulating evidence suggest that the enteric nervous system (ENS) plays important roles in gastrointestinal inflammatory responses, which could be in part mediated by Toll-like receptor (TLR) activation. The aim of this study was to characterise the expression and functionality of TLR2/4/9 in the ENS.

**Methods:**

TLR2/4/9 expression was assessed in the plexuses of adult rats and embryonic ENS cultures by immunofluorescence and quantitative PCR. Following stimulation with TLR2/4/9 ligands or their combinations, activation of NF-kB, production of TNF-α, IL-6 and MCP-1 and chemoattraction of RAW264.7 macrophages were evaluated by means of Western blot, ELISA, immunofluorescence and migration assays in transwell inserts.

**Results:**

TLR2/4/9 staining colocalised with enteric neuronal markers, whereas their presence in enteroglial processes was low to inexistent. Stimulation of ENS cultures with selective ligands induced NF-kB activation and release of cytokines and chemokines by neurons and resident immunocytes. TLR2 neutralisation before lipopolysaccharide (LPS) challenge reduced production of inflammatory mediators, whereas combination of TLR2/4 ligands promoted macrophage migration. Combined stimulation of cultures with LPS and the CpG oligonucleotide 1826 (TLR4/9 ligands) caused a synergic increase in chemoattraction and cytokine production.

**Conclusions:**

Our results suggest that the ENS, and particularly enteric neurons, can integrate a variety of microbial signals and respond in a relatively selective fashion, depending on the particular TLRs stimulated. These findings additionally suggest that the ENS is capable of initiating a defensive response against pathogens and expanding inflammation.

**Electronic supplementary material:**

The online version of this article (doi:10.1186/s12974-016-0653-0) contains supplementary material, which is available to authorized users.

## Background

The enteric nervous system (ENS) is the largest component of the peripheral nervous system and is constituted by two cell types, neurons and enteric glial cells (EGC). It is organized in two major ganglionated plexuses, namely the Meissner’s submucosal (SMP) and the Auerbach’s myenteric (LMMP) plexuses, which control gastrointestinal motility, secretion and blood flow, participate in maintenance of the epithelial barrier and modulate various processes of the local immune response [1, 2]. Although a number of studies have demonstrated that the ENS undergoes structural and phenotypic plastic changes during inflammatory responses [3], growing evidence suggests that it is not only a bystander, but an active player during inflammation. Indeed, both enteric neurons and EGCs have been shown to contribute to the resulting inflammatory phenotype following chemically induced colitis [4, 5], probably through the release of several immune mediators [6–9]. Moreover, perineural inflammation is dense in biopsies from inflammatory bowel disease patients and is correlated to major histocompatibility complex class II expression in EGCs [10], further illustrating the neuroimmune interactions existing in the intestine.

Toll-like receptors (TLR) are transmembrane receptors that recognise different highly conserved microorganism-associated molecular patterns (MAMP), as well as other molecules such as damage-associated molecular patterns [11, 12]. Upon MAMP binding, the cytoplasmic domain recruits different adapter proteins to trigger a variety of signalling pathways that ultimately activate transcription factors such as nuclear factor-kB (NF-kB), activating protein-1 and interferon regulatory factors, which in turn promote the production of pro-inflammatory cytokines [13, 14]. TLRs are expressed in most human tissues including the gastrointestinal tract [15], where they have been proposed to mediate the cross-talk between host cells and commensal microflora due to the key role they play in the innate immune response and in shaping adaptive immunity [16, 17].

In recent years, expression of some TLRs in the ENS has been described [18, 19], and their roles in intestinal motility, apoptosis and normal development have progressively been unravelled [19, 20]. In addition, their immune functions have been also addressed by some authors [7, 21], but there are still several questions to be answered, such as whether differential recognition of MAMPs by the ENS translates into microbial-selective responses, or whether TLR-mediated signalling is involved in expansion of the inflammatory response. In this context, we aimed to characterise expression and functionality of TLR2/4/9 in the ENS, focusing on their responses in terms of cytokine, chemokine and chemoattraction induction after single or combined ligand challenges. Our results show that neurons are the main TLR-expressing cells in enteric plexuses and integrate TLR signals promoting diverse responses depending on the stimulus received, bringing some new evidence regarding their roles in defence against pathogens and inflammation enhancement.

## Methods

### Reagents and antibodies

All culture media, foetal bovine serum, antibiotics, N-2 supplement and 4′,6-diamidino-2-phenylindole (DAPI) were from Life Technologies (El Prat de Llobregat, Spain). Trypsin, DNase I, gelatin and Bay 11-7082 were from Sigma (Madrid, Spain). The synthetic diacylated lipopeptide Pam2CSK4, a TLR2/6-specific agonist, was purchased from InvivoGen (San Diego, USA). Lipopolysaccharide (LPS) stimulation of TLR4 was performed with a mixture 1:1 of LPS from *Escherichia coli* O55:B5 and *Salmonella typhosa*, both purchased from Sigma. Phosphorothioate-modified class B CpG oligonucleotides (ODN) 1826 5′-TCCATGACGTTCCTGACGTT-3′ and 1826 control (cODN) 5′-TCCATGAGCTTCCTGAGCTT-3′ synthesised by Tib-Molbiol (Berlin, Germany) were used to stimulate TLR9. Primary antibodies used in immunofluorescence were rabbit monoclonal anti-TLR2 (1:500; Abcam, Cambridge, UK), rabbit polyclonal anti-TLR4 (1:100; Novus Biologicals, Cambridge, UK), mouse monoclonal anti-TLR9 (1:100; Novus Biologicals), chicken polyclonal anti-GFAP (1:500; Antibodies-online, Aachen, Germany), mouse monoclonal anti- S100β (1:1000; Abcam), mouse monoclonal anti-HuC/D (1:200; Life Technologies), chicken polyclonal anti-β-tubulin III (1:500; Abcam), rabbit monoclonal anti-NF-kB p65 (1:50; Cell Signaling Technology, Danvers, USA), goat polyclonal anti-IL-6 (1:250; Santa Cruz Biotechnology), mouse monoclonal anti-MCP-1 (1:100; Novus Biologicals) and rabbit polyclonal anti- ionized calcium-binding adapter molecule (IBA)-1 (1:500; Wako Chemicals). Secondary antibodies used were Alexa Fluor 488 and 568 donkey and goat anti-rabbit IgG, respectively; Alexa Fluor 568 goat anti-mouse IgG and Alexa Fluor 647 donkey anti-mouse IgG (1:500; all from Life Technologies), CF488A donkey anti-chicken IgY (1:2000) and CF488A donkey anti-mouse IgG (1:500; both from Biotium, Hayward, USA). For Western blotting, rabbit monoclonal anti-phospho-IkBα (1:1000; Cell Signaling Technology), mouse monoclonal anti-β-actin (1:5000; Sigma), horseradish peroxidase (HRP)-linked goat anti-rabbit IgG (1:10,000; Cell Signaling Technology) and HRP-linked sheep anti-mouse IgG (1:100,000; GE Healthcare, Barcelona, Spain) antibodies were used. TLR2 neutralisation was performed with a mouse monoclonal anti-TLR2 antibody (clone T2.5, 10 μg/mL; Novus Biologicals).

### Animals

For ex vivo experiments, 10-week-old male Sprague-Dawley rats were purchased from Charles River (Les Oncins, France) and housed in specific pathogen-free conditions, under a controlled temperature (20 ± 2 °C) and photoperiod (12 h/12 h light-dark cycle), with free access to food and water. Animals were euthanized by CO_2_ inhalation and distal ileum was removed and placed in ice-cold oxygenated Krebs solution for subsequent manipulation. Colons were flushed with Krebs solution, opened along the mesenteric border, pinned flat in a dissection dish and fixed in Lana’s fixative (4 % paraformaldehyde, 14 % picric acid in 0.4 M phosphate buffer).

For in vitro experiments, pregnant Sprague-Dawley rats purchased from Charles River were killed by CO_2_ inhalation followed by cardiac puncture exsanguination. Pregnant uteri were removed and kept in ice-cold phosphate-buffered saline (PBS) for further dissection.

All animal procedures performed were approved by the Ethical Committee of the Universitat Autònoma de Barcelona (code 2669).

### Cell cultures

Isolation and culture of rat embryonic ENS was performed as described elsewhere [22]. Briefly, intestines of rat embryos (E16) were removed and finely diced in PBS. Tissue fragments were digested with trypsin and DNase I, and cells obtained were counted and seeded at a density of 2.4 × 10^5^ cells/cm^2^ on 24- or 48-well plates, previously coated with a 0.5 % gelatin solution in sterile PBS. Stimulation was performed for 24 h after 15-day culture in serum-free medium (DMEM-F12 (1:1)) containing 1 % of N-2 supplement.

The murine macrophage cell line RAW 264.7 (ATCC® TIB-71) was purchased from the American Type Culture Collection and cultured in DMEM supplemented with 10 % heat-inactivated foetal calf serum.

Stimulation experiments were performed for 24 h with either 100 ng/mL Pam2CSK4, 100 ng/mL LPS, 1 μM ODN 1826, 1 μM cODN 1826 or combinations of these ligands. For NF-kB inhibition and TLR2 neutralisation experiments, cultures were pre-treated for 1 h with 15 μM Bay 11-7082 or 10 μg/mL anti-TLR2 antibody before MAMP stimulation.

In costimulation experiments, comparison between expected additive effects and measured effects of TLR ligand combinations was calculated according to the model of functional interaction, represented by the following equation as described in [23]: E(ODN + LPS)expected = E(ODN)measured + E(LPS)measured – E(ODN)measured * E(LPS)measured.

### Immunofluorescence

Fixed colon was dissected under a stereo microscope to obtain whole-mount preparations of the SMP and the LMMP. Adult tissues, as well as ENS cultures grown on cover-slips, were blocked for 1 h in PBS containing 4 % horse serum, 0.1 % Triton X-100 and 0.01 % NaN_3_. Samples were incubated overnight at 4 °C with combinations of TLR2, TLR4 or TLR9 with Hu C/D, β-tubulin III (neuronal markers), GFAP, S100β (glial markers), IL-6, MCP-1 or IBA-1 antibodies. Secondary antibodies to rabbit, mouse or goat IgG and chicken IgY were used to detect bound primary antibodies. All samples were mounted in Vectashield aqueous anti-fading mounting medium (Vector Laboratories) and analysed under a Zeiss LSM 700 confocal laser microscope (Carl Zeiss, Madrid, Spain). To avoid overlapping, control preparations were previously single stained for one marker, and their fluorescence was evaluated with the three different lasers used for final image acquisition. Band-pass filters were set up to avoid cross-talk between channels, and sequential acquisition was performed during experiments to excite fluorochromes one at a time.

### Real-time RT-PCR analysis

Total RNA from ENS culture was extracted using the RNeasy Mini Kit (QIAGEN, Las Matas, Spain), quantified by optical densitometry and assessed for integrity by on-chip gel electrophoresis with the Experion™ System (Bio-Rad Laboratories, el Prat de Llobregat, Spain). Then, 100 ng of RNA were retro-transcribed by using the Transcriptor First-strand cDNA Synthesis Kit (Roche Applied Science, Mannheim, Germany) for reverse-transcriptase polymerase chain reaction (RT-PCR). Primer sequences listed in Table 1 were designed to span introns using the Universal ProbeLibrary Assay design Center (https://lifescience.roche.com/webapp/wcs/stores/servlet/CategoryDisplay?tab=Assay+Design+Center&identifier=Universal+Probe+Library&langId=-1), and checked for specificity through BLAST search. PCR amplifications were performed using the LC480 SYBR Green I Mastermix (Roche Applied Science) according to manufacturer’s protocol, and run on a LightCycler 480 II instrument (Roche Applied Science). Absence of coamplification products was assured by generating a final melting curve for each reaction and by loading PCR products on a denaturing 2 % agarose gel, stained with SYBR safe (Life Technologies) and visualized under UV transillumination. Specificity of the primers was also determined by sequencing these amplification products. Messenger RNA (mRNA) level of expression of the genes of interest was corrected to that of the S6 housekeeping gene and calculated by the ΔΔCt method.

**Table 1:** List of primers used for real-time RT-PCR analysis

Genes	Sense primer	Antisense primer	Reference
*rS6*	CCAAGCTTATTCAGCGTCTTGTTACTCC	CCCTCGAGTCCTTCATTCTCTTGGC	NM_017160
*rTLR2*	CAGATGGCCAGAGGACTCA	AATGGCCTTCCCTTGAGAG	ENSRNOT00000013025.3
*rTLR4*	GGATGATGCCTCTCTTGCAT	TGATCCATGCATTGGTAGGTAA	NM_019178.1
*rTLR9*	TCCGTGACAATCACCTCTCTT	GGTCCAGGTCTCGCAGATT	NM_198131.1

In order to compare mRNA expression levels of the receptors in basal conditions, absolute mRNA levels were estimated by determining the difference between the cycle threshold (Ct) of the target receptor and the Ct of the housekeeping gene, as described elsewhere [21, 24]. According to their ΔCt to the S6 gene, genes were classified as high-expressed (ΔCt less than 5 cycles), intermediate-expressed (ΔCt from 5 to 15 cycles), low or rare-expressed (ΔCt superior to 15 cycles) and undetectable (ΔCt superior to 40 cycles).

### Western blot

ENS cultures were harvested in RIPA lysis buffer (Millipore, Madrid, Spain) containing 2 mM sodium orthovanadate, phosphatase inhibitor cocktail 3 (Sigma) and a tablet of Complete™ protease inhibitors cocktail (Roche Applied Science). Protein samples (30 μg) were separated on a 10 % acrylamide gel containing 0.1 % sodium dodecyl sulfate and transferred to a nitrocellulose membrane with the iBlot™ Dry Blotting System (Life Technologies). Membranes were blocked for 1 h at room temperature with 5 % non-fat dry milk in Tris-buffered saline (100 mM NaCl, 10 mM Tris, pH 7.5) with 0.1 % Tween 20 (TBST), and incubated overnight at 4 °C with primary antibodies in a 5 % BSA solution in TBST. Bound antibodies were detected with HRP-conjugated anti-rabbit or anti-mouse antibodies, and visualized by enhanced chemiluminescent detection (ECL advance, GE Healthcare). Membranes were stripped for 15 min in Reblot buffer (Millipore), followed by extensive washing in TBST before reblocking with 5 % non-fat dry milk in TBST and reprobing for β-actin determination. Bands were imaged in a LAS-3000 Imager (Fujifilm, Tokyo, Japan) and quantified with Multigauge 3.0 software (Fujifilm). To allow comparison between different membranes, the density of the bands was referred to that of untreated controls and normalized to the amount of β-actin in the same sample.

### TNF-α, IL-6 and MCP-1 ELISA

Culture supernatants were centrifuged, aliquoted and frozen, and further assayed with the corresponding BD OptEIA™ ELISA Sets (BD), following manufacturer’s recommended assay procedures. Final cytokine or chemokine values were related to the total protein amount of the sample, which was determined by using the BCA protein assay kit (Pierce, Rockford, USA).

### Migration assays

Twenty-four hours after stimulation of ENS primary culture with MAMPs, conditioned supernatants were centrifuged, placed into 24-well plates and left to equilibrate for an hour with the transwell insert. Then, 10^5^ RAW 264.7 macrophages were seeded in the upper chamber of the 8-μm-pore transwell inserts, and allowed to migrate for 4 h at 37 °C and 5 % CO_2_. After fixation in 4 % paraformaldehyde, cells on the upper surface of the transwell membrane were removed by rubbing with a sterile cotton swab, and cells on the lower surface were stained with DAPI. The average number of migrating cells was determined by counting eight fields per membrane at ×100 under a Carl Zeiss Axioskop 40 FL epifluorescence microscope equipped with a Zeiss AxioCam MRm camera (Carl Zeiss, Germany). Each experiment was performed in duplicate.

### Statistical analysis

Results are presented as mean values ± S.E.M. of at least three independent experiments. All data were compared using one-way or two-way ANOVA, followed by Tukey’s post hoc test (unless otherwise stated). Where stated, randomised block design analysis was performed to minimise the variability due to differences between individual culture responses. Data analysis and plot were performed with GraphPad Prism 5.0 software (GraphPad Software Inc., La Jolla, USA). Randomised block design analyses were performed with Minitab 15 Statistical Software (Minitab Inc., Pennsylvania, USA). A *P* value <0.05 was considered to be significant.

## Results

### Enteric neurons express TLR2/4/9 in adult rat colon

**Fig. 1:**
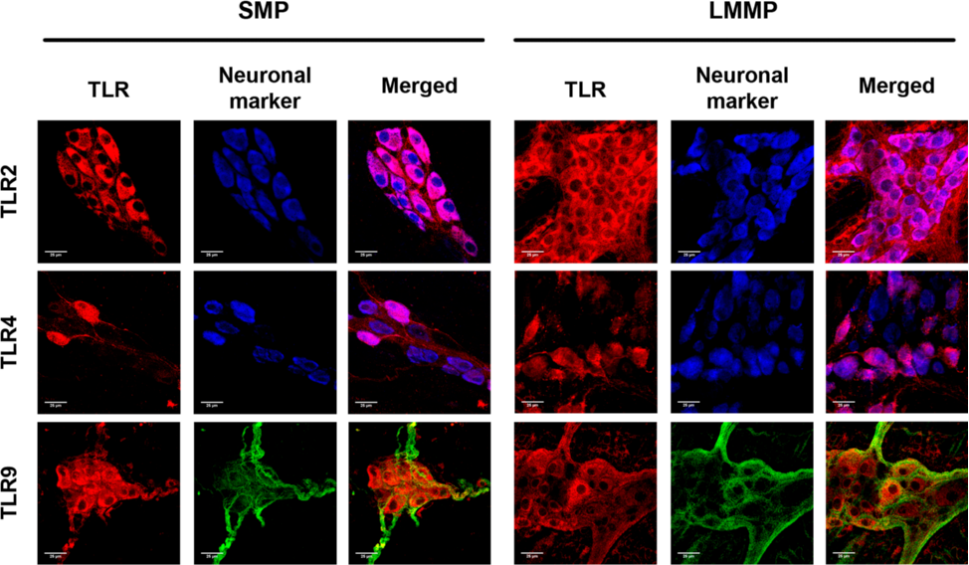
TLR2/4/9 are expressed in enteric neurons of adult rat colon tissue. TLR localisation in whole-mount preparations of the SMP and the LMMP. B-tubulin III was used instead of HuC/D as neuronal marker for colocalisation with TLR9 due to antibody detection incompatibilities. *Scale bars*: 25 μm

Expression of TLR2/4/9 in whole-mount preparations from adult rat colon was assessed by immunofluorescence. Prominent immunoreactivity for the three receptors was found in neurons from both SMP and LMMP (Fig. 1), as demonstrated by colocalisation with the neuronal markers Hu C/D or β-tubulin III. However, distribution patterns were different for each receptor. TLR2 staining was found in all neurons of the SMP and the LMMP (Fig. 1, upper panels), whereas TLR4 was only observed in discrete neuronal somas and fibres (Fig. 1, middle panels, and Additional file 1). TLR9 reactivity was found in neurons and interganglionic bundles, fully colocalising with the β-tubulin III marker (Fig. 1, lower panels).

Since expression of TLR2 and 4 has previously been described in EGCs [18, 19, 21], we additionally performed colocalisation studies of the three receptors with the enteroglial marker glial fibrillary acidic protein (GFAP). Our results demonstrate minor localisation of the TLRs studied in EGCs in the SMP (Additional file 2, left panels). In the same vein, TLR4 and 9 showed minor colocalisation with GFAP^+^ cells in the LMMP (Additional file 2, right middle and lower panels). Conversely, TLR2 immunoreactivity was observed in some enteroglial processes in the LMMP (Additional file 3), indicating that this receptor is expressed in both neurons and EGCs.

### TLR2/4/9 show similar expression patterns in rat embryonic ENS culture

**Fig. 2:**
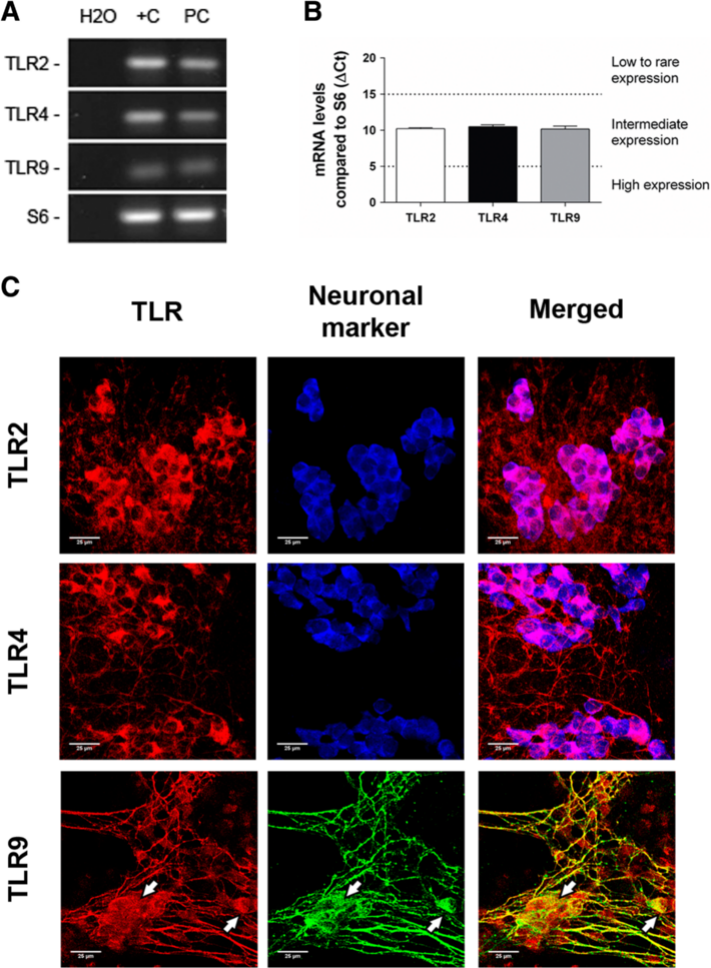
Embryonic ENS culture neurons express TLR2/4/9. **a** Agarose gel showing specific products of real-time PCR for the assayed genes in ENS culture (PC); water was used as a no-template control (H_2_O) and rat colon cDNA as positive control (+C). **b** TLR relative expression in ENS culture in basal conditions (*n* = 8). **c** Localisation of TLR in ENS culture ganglia. B-tubulin III was used instead of HuC/D as neuronal marker for colocalisation with TLR9 due to antibody detection incompatibilities. *White arrows* point to neuronal somas. *Scale bars*: 25 μm

To determine whether rat embryonic ENS cultures might be a good in vitro model to study the immune functions of TLRs, we characterised their expression in such set-up. The three receptors were found in ENS cultures, displaying similar expression levels (Fig. 2a, b). Distribution of such receptors was circumscribed to neuronal structures, either somas or axons. TLR2 was found in all neurons (Fig. 2c, upper panels), whereas TLR4 was seen in most but not all somas, as well as in discrete fibres (Fig. 2c, middle panels). TLR9 positive staining colocalised with β-tubulin III^+^ structures, including neuronal somas (arrows) and nerve bundles (Fig. 2c, lower panels).

### Microbial motifs induce TLR-mediated activation of the NF-kB signalling pathway

Recognition of MAMPs through TLRs leads to activation of different signalling cascades, including the NF-kB pathway. Phosphorylation of the inhibitor of kB (IkB)-α protein is necessary to target it for ubiquitination and release NF-kB subunits, which are then able to translocate to the nucleus [14]. Therefore, p-IkBα was used as an indicator of TLR-induced activation of ENS cultures.

**Fig. 3:**
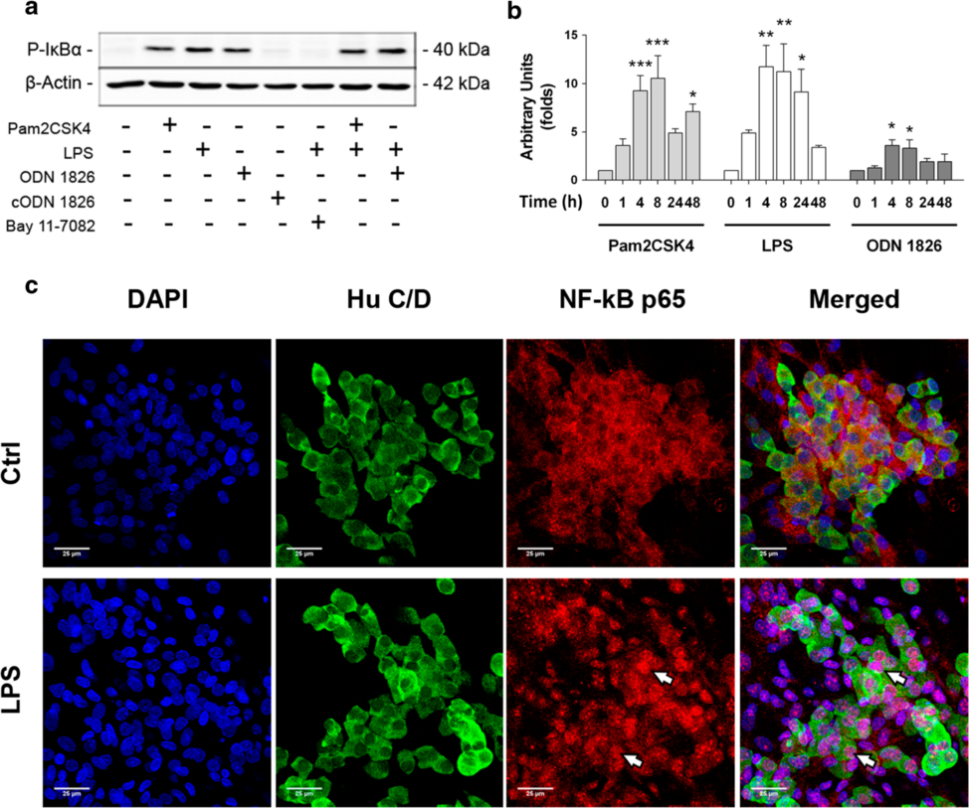
MAMP stimulation induces activation of the NF-kB pathway in embryonic ENS culture. **a** Rat embryonic ENS culture was incubated for 8 h with the indicated MAMPs or the IkB phosphorylation inhibitor Bay 11-7082, and cell protein extraction and Western blot were performed to determine phosphorylated IkB (P-IkBα). B-actin was used as a loading control. **b** Time-course densitometric quantification of P-IkBα bands corrected to β-actin and related to basal activation levels; representative bands are shown in Additional file 4. Statistical analysis was performed independently for each ligand, using one-way ANOVA followed by Dunnett’s test (*n* = 4 for each ligand and time point; *P < 0.05, **P < 0.01 and ***P < 0.001). **c** Representative micrographs showing NF-kB p65 subunit localisation in basal conditions (Ctrl) or 1 h after LPS challenge (LPS). *White arrows* point to neuronal nuclei displaying p65 positive staining. *Scale bars*: 25 μm

The three TLRs described were functional, as the ligands Pam2CSK4 (TLR2/6), LPS (TLR4) and ODN 1826 (TLR9) caused a time-dependent increase in phosphorylation of this protein (Fig. 3a, b). Furthermore, the cODN 1826, which does not bear the CG motifs, did not induce NF-kB activation, demonstrating the selectivity of the TLR9 ligand. Pre-incubation of ENS cultures with 15 μM Bay 11-7082 abrogated LPS-induced activation, confirming the specific involvement of the NF-kB pathway (Fig. 3a).

Activation kinetics observed for all MAMPs were similar, lasting from 1 to 48 h and peaking at 4–8 h (Fig. 3b). However, activation intensity was ligand-specific, LPS being the most potent inducer (LPS_0.1_ = 386.6 ± 61.8, Pam2CSK4_100_ = 328.3 ± 29.14 and ODN 1826_1_ = 110 ± 15.98 AUxhour; Additional file 4A-E).

Neuronal activation was determined through immunofluorescent localisation of the NF-kB p65 subunit. Unstimulated ENS culture showed cytoplasmic staining for p65 in neurons and other undetermined cell types (Fig. 3c, upper panels). However, 1 h after LPS stimulation, p65 reactivity was preferentially seen in nuclear localisation, colocalising with DAPI. Interestingly, some of the TLR4-activated cells were HuC/D^+^ neurons (Fig. 3c, lower panels), indicating that TLRs expressed in these cells signal through the NF-kB pathway.

### LPS stimulation induces a pro-inflammatory microenvironment in ENS culture through the NF-kB signalling cascade

Tumour necrosis factor (TNF)-α and interleukin (IL)-6 are secreted in rat embryonic ENS culture in response to LPS [7]. In addition, IL-8 is also released in vitro by LPS-stimulated jejunum biopsies [25]. Therefore, we assessed the production of TNF-α, IL-6 and the monocyte chemoattractant protein (MCP)-1 upon TLR2/4/9 ligand challenge, as well as the involvement of the NF-kB pathway in these responses.

**Fig. 4:**
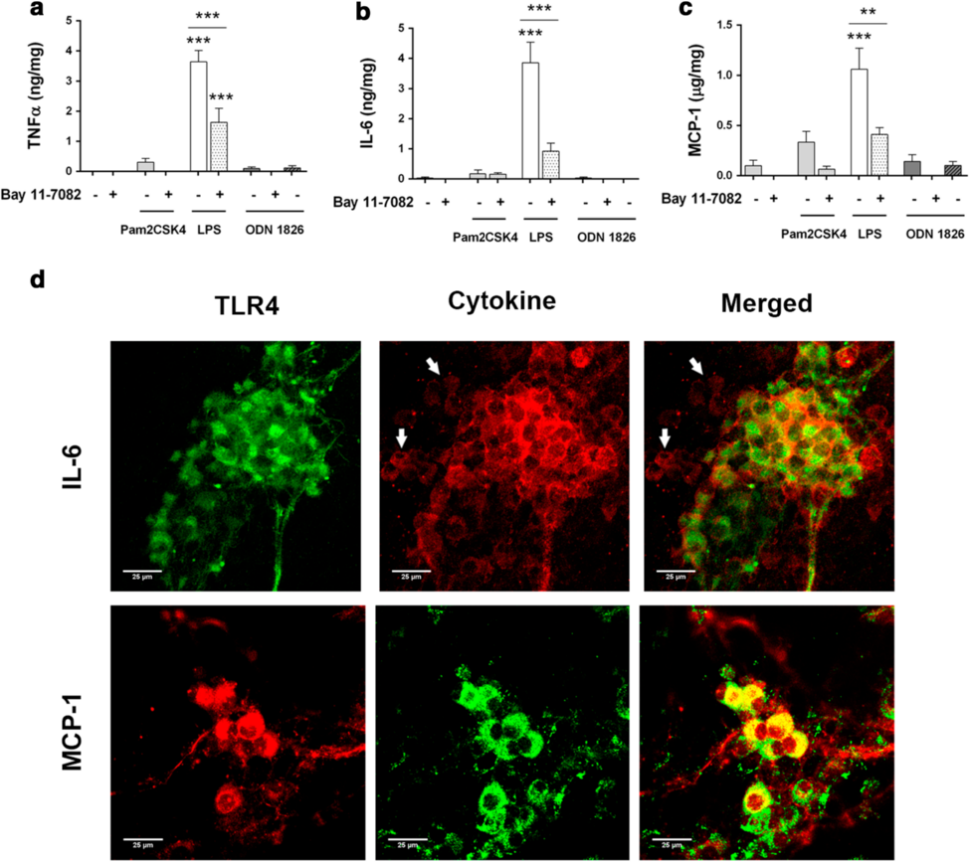
ENS culture releases cytokines and chemokines in response to LPS. Rat embryonic ENS culture was stimulated for 24 h and culture supernatants were collected and centrifuged prior to measuring cytokine and chemokine secretion. **a** TNF-α (*n* = 4–8; LPS vs. control and Bay 11-7082 + LPS, ***P < 0.001; Bay 11-7082 + LPS vs. control, ***P < 0.001). **b** IL-6 (*n* = 4–8; LPS vs. control and Bay 11-7082 + LPS, ***P < 0.001). **c** MCP-1 (*n* = 4–8; LPS vs. control, ***P < 0.001; LPS vs. Bay 11-7082 + LPS, **P < 0.01). **d** Distribution studies show IL-6 and MCP-1 colocalisation with TLR4^+^ neurons. *White arrows* show TLR4^-^ IL-6-producing cells. *Scale bars*: 25 μm

LPS addition to embryonic ENS culture elicited a marked increase in all inflammatory mediators analysed 24 h after stimulation (Fig. 4a–c). In contrast, the TLR2/6 ligand Pam2CSK4 induced a slight, though not significant, increase in TNF-α and MCP-1, whereas ODN 1826 was not able to induce secretion of such molecules (Fig. 4a–c). These results suggest that Gram-negative signalling induces pro-inflammatory responses in embryonic ENS culture, while Gram-positive structures and bacterial DNA do not, despite their ability to activate the NF-kB pathway.

Inhibition of the NF-kB pathway by Bay 11-7082 significantly decreased the production of all studied mediators, but did not completely abrogate it (Fig. 4a–c). This observation points out that other signalling pathways, such as the mitogen-activated protein kinases (MAPK) cascade [7], might also be involved in responses to these MAMPs.

TNF-α expression after LPS challenge has been described in embryonic ENS neurons [7]. Since TLR4 staining in ENS cultures was found circumscribed to neurons, we sought to determine whether TLR4^+^ cells were responsible for the production of IL-6 and MCP-1 24 h after LPS challenge. Colocalisation studies revealed that TLR4-expressing neurons produced IL-6 (Fig. 4d, upper panels) and MCP-1 (Fig. 4d, lower panels). However, macrophages did also show immunoreactivity to IL-6 (Fig. 4d, upper panels, white arrows, and Additional file 5) and MCP-1 antibodies (Additional file 6, white arrows), pointing out that resident immunocytes are also involved in responses to MAMPs in these cultures. Nevertheless, neurons were the only IL-6-producing cells that stained positive for TLR4 (Additional file 7), suggesting they act as primary sensors of LPS in these cultures.

### TLR2 is up-regulated upon stimulation with TLR2/4/9 ligands

**Fig. 5:**
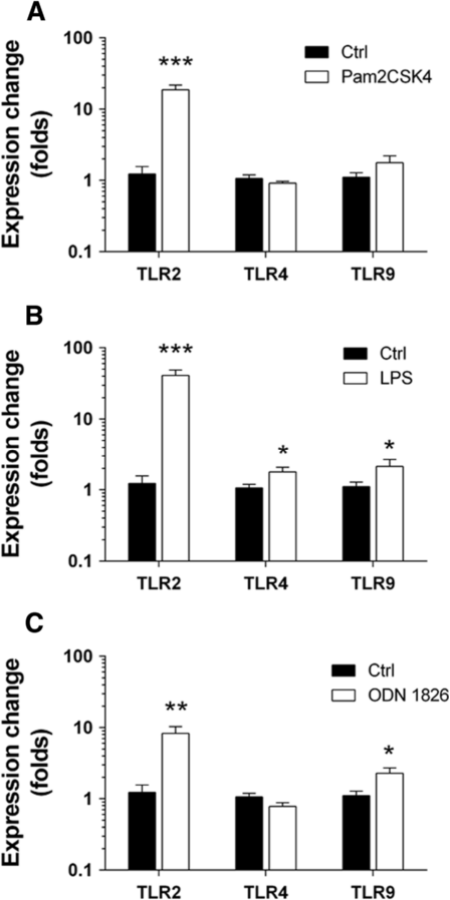
TLR activation in ENS culture is associated to TLR2 up-regulation. TLR mRNA levels were assessed in rat ENS culture stimulated for 8 h with the indicated MAMPs. **a** Pam2CSK4 (*n* = 5–10; TLR2 ligand vs. control, ***P < 0.001). **b** LPS (*n* = 5–10; TLR2 ligand vs. control, ***P < 0.001; TLR4 and TLR9 ligand vs. control, *P < 0.05). **c** ODN 1826 (*n* = 5–10; TLR2 ligand vs. control, **P < 0.01; TLR9 ligand vs. control, *P < 0.05)

We next aimed to evaluate whether stimulation with one particular MAMP might have cross-regulatory effects on the expression of other TLRs, as has been previously described [26, 27]. Quantitative PCR assays of ENS culture showed consistent TLR2 up-regulation after an 8-h exposure to the tested MAMPs (TLR2_Pam2CSK4_ = 18.78 ± 3.02, TLR2_LPS_ = 40.78 ± 7.84 and TLR2_ODN 1826_ = 8.29 ± 2 vs. TLR2_Ctrl_ = 1.23 ± 0.33 folds; Fig. 5a–c). Moreover, a slight increase in TLR4 (TLR4_LPS_ = 1.78 ± 0.28 vs. TLR4_Ctrl_ = 1.06 ± 0.13 folds; Fig. 5b) and TLR9 expression (TLR9_LPS_ = 2.13 ± 0.53 vs. TLR9_Ctrl_ = 1.1 ± 0.17 folds; Fig. 5b) was observed after LPS challenge. Finally, ODN 1826 stimulation did also up-regulate TLR9 expression (TLR9_ODN 1826_ = 2.28 ± 0.43 vs. TLR9_Ctrl_ = 1.1 ± 0.17 folds; Fig. 5c).

### Neutralisation of TLR2 modulates TLR4-mediated production of inflammatory mediators

**Fig. 6:**
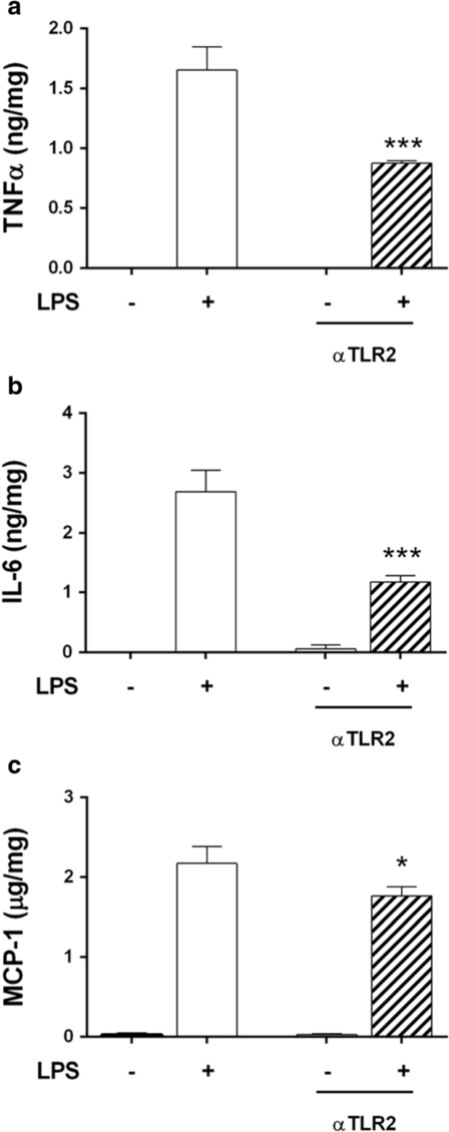
TLR2 blockade reduces ENS culture responses to LPS. Embryonic ENS culture was pre-incubated for 1 h with 10 μg/mL of TLR2 neutralising antibody before an LPS challenge for 24 additional hours. **a** TNF-α (*n* = 3; LPS vs. αTLR2 + LPS, ***P < 0.001). **b** IL-6 (*n* = 3; LPS vs. αTLR2 + LPS, ***P < 0.001). **c** MCP-1 (*n* = 3; LPS vs. αTLR2 + LPS, *P < 0.05)

Consistent overexpression of TLR2 after MAMP challenge prompted us to study the involvement of this receptor in LPS-elicited pro-inflammatory responses. Pre-incubation with a TLR2-neutralising antibody significantly decreased the release of TNF-α, IL-6 and MCP-1 (Fig. 6a–c). These results point out that TLR2 shapes TLR4-driven responses to its ligand LPS, increasing their final effect.

### TLR4/9 synergise amplifying the pro-inflammatory responses elicited by LPS

Given that the described alterations in TLR expression might influence subsequent responses to LPS, we studied the production cytokines and chemokines after combined stimulation with the selected MAMPs.

**Fig. 7:**
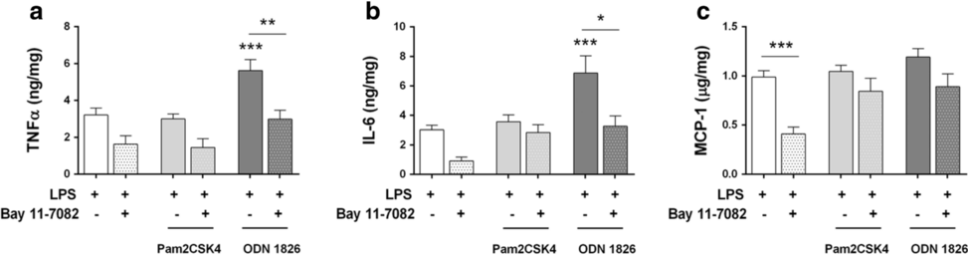
TLR4/9 combined stimulation elicits synergic pro-inflammatory responses in ENS culture. Embryonic ENS cultures were incubated with the specified combinations of MAMPs for 24 h. Randomised block design analysis followed by Tukey’s post hoc test was applied to minimise statistical differences due to intrinsic culture responsiveness. **a** TNF-α (*n* = 4–9; ODN 1826 + LPS vs. LPS, ***P < 0.001; ODN 1826 + LPS vs. Bay 11-7082 + ODN 1826 + LPS, **P < 0.01). **b** IL-6 (*n* = 4–9; ODN 1826 + LPS vs. LPS, ***P < 0.001; ODN 1826 + LPS vs. Bay 11-7082 + ODN 1826 + LPS, *P < 0.05). **c** MCP-1 (*n* = 4–9; LPS vs. Bay 11-7082 + LPS, ***P < 0.001)

Since TLR2 is strongly up-regulated after LPS exposure, we expected that costimulation with Pam2CSK4 would increase cytokine and chemokine production. However, addition of this ligand to LPS-challenged ENS culture did not induce a higher mediator release (Fig. 7a–c). Nevertheless, the inhibitory effects of Bay 11-7082 in production of IL-6 and MCP-1 were reverted by this combined treatment (Fig. 7b, c), suggesting that Pam2CSK4 + LPS incubation might involve activation of signalling pathways other than NF-kB.

On the other hand, simultaneous addition of TLR4 and TLR9 ligands (LPS and ODN 1826) caused a synergistic increase in production of TNF-α and IL-6 in embryonic ENS cultures (Fig. 7a, b; Table 2), and reduced the inhibitory effects of Bay 11-7082 in MCP-1 production (Fig. 7c).

**Table 2:** Comparison between expected additive effects and measured effects of TLR ligand interactions in embryonic ENS culture

Cytokine	TLR ligand combination	Expected additive effect (%)	Measured effect (%)	*N*	*P*
TNF-α	Pam2CSK4 + LPS	53.9 ± 8.5	52.1 ± 7.8	5	0.744
	ODN 1826 + LPS	51.9 ± 8.5	100 ± 20.4	5	0.02
IL-6	Pam2CSK4 + LPS	43.7 ± 5.7	45 ± 8.5	5	0.729
	ODN 1826 + LPS	42.3 ± 5.9	100 ± 24.8	5	0.048
MCP-1	Pam2CSK4 + LPS	91.5 ± 3.6	85.2 ± 5.3	5	0.102
	ODN 1826 + LPS	88.2 ± 4.3	100 ± 5.4	5	0.02

The mean production of TNF-α, IL-6 or MCP-1 elicited by ODN 1826 + LPS was considered as 100 % effect. The expected additive effect of the combination of two ligands was calculated according to the model of functional interaction, represented by the equation E_(ODN+LPS)expected_ = E_(ODN)measured_ + E_(LPS)measured_ – E_(ODN)measured_ * E_(LPS)measured_. Statistical comparisons were performed by means of randomised block design analysis to minimise the random effects due to intrinsic culture responsiveness

### TLR2/4 and TLR4/9 interact enhancing chemoattraction of a macrophage cell line

The release of MCP-1 regulates migration and infiltration of different cell types, especially monocytes/macrophages [28]. In addition, other chemokines might be secreted in ENS culture upon stimulation with MAMPs [29]. Therefore, we finally aimed to study whether the supernatants of challenged ENS primary cultures could induce the recruitment of immune cells.

**Fig. 8:**
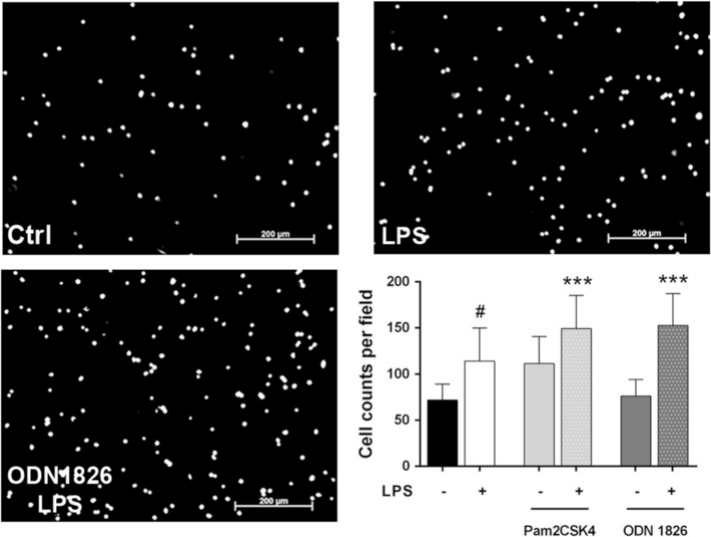
Stimulated ENS culture conditioned media induce chemoattraction of RAW 264.7 macrophages. RAW 264.7 macrophages were seeded in the upper chamber of 8-μm-pore transwell inserts and left to migrate for 4 h towards lower chambers filled with stimulated ENS culture conditioned media. Representative micrographs of the evaluated fields in the lower chamber of a transwell insert mounted upon unstimulated (Ctrl), LPS-stimulated (LPS) or ODN 1826 + LPS-stimulated (ODN 1826 + LPS) ENS culture medium. *Scale bars*: 200 μm. Number of migrating cells per field (*n* = 4, with two replicates for experiment; LPS vs. unstimulated ENS culture supernatant, #*P* = 0.08; Pam2CSK4 + LPS and ODN 1826 + LPS vs. unstimulated ENS culture supernatant, ***P < 0.001). Randomised block design analysis was performed to minimise the variability in migration due to RAW 264.7 passage, followed by Tukey’s post hoc test

MAMP-enriched DMEM culture medium did not induce RAW 264.7 cell migration to the lower compartment of the transwell inserts (data not shown). Similarly, conditioned medium from non-stimulated ENS culture did not increase migration when compared to DMEM culture medium (data not shown). However, conditioned medium obtained from LPS-stimulated ENS culture showed a tendency to increase migration to the lower chamber (59 % increase, *P* = 0.08; Fig. 8). This chemotactic effect was further enhanced to a 107 and 112 % increase by media conditioned by a combination of Pam2CSK4 or ODN 1826 with LPS, respectively (*P* < 0.001 for both, Fig. 8). Taken together, these data suggest that MAMPs themselves are not chemoattractant, but LPS alone or in combination with other TLR ligands elicits secretion of chemotactic substances by enteric neurons and resident immunocytes, promoting migration of RAW 264.7 macrophages.

## Discussion

Since their initial characterisation in human in the late 90s [13], TLRs have been widely described in several tissues and cells, including central and peripheral nervous systems [15, 30]. In recent years, the study of their role in the ENS has gained attention; however, there are still only a few works addressing their participation in the immune response to microbes. In this study, we report the expression and functionality of TLR2/4/9 in embryonic ENS primary cultures, emphasising in their interactions in terms of cytokine and chemokine production. Our results indicate that enteric neurons respond to MAMPs through secretion of inflammatory mediators, integrating their signals to trigger a tailored response to each challenge. Indeed, activation of TLR2 or TLR9 upon LPS stimulation enhanced macrophage chemoattraction, while potentiating different pro-inflammatory responses. Taken together, these findings suggest that TLRs confer the ENS the ability to discriminate microbial signals and expand inflammation by promoting particular pro-inflammatory microenvironments and interacting with resident immunocytes to chemoattract immune cells. These facts further support the idea that the ENS is an immunologically active tissue.

Expression of TLRs within the ENS has been previously assessed by other groups in full-thickness tissue sections of the small intestine [18, 25]. In this regard, colocalisation studies in SMP and LMMP whole-mounts from adult rat colon offer an improved perspective to discern immunoreactive cell populations. Our findings in these tissues confirm previous data about TLR2 expression, which had been described in both HuC/D^+^ and GFAP^+^ cells [19]. Conversely, we could only observe TLR4 staining in some subsets of enteric neurons [20, 25], even though expression in EGCs has been already reported [18, 21]. In this regard, our results agree with parallel studies in central nervous system cultures pointing out that neurons have more prominent TLR4 expression than astrocytes [29]. Finally, the TLR9 expression patterns we report here match with previous work that localise this receptor in neuronal somas and β-tubulin III^+^ microtubules from dorsal root ganglia cultures [31] and various brain sections [32]. Interestingly, TLR distribution in embryonic ENS primary cultures was very similar to that of adult tissues, which might indicate that few modifications affect their localisation during development. Indeed, minor TLR2 and TLR4 expression variations have been observed in the mouse developing brain, whereas TLR9 increases in adulthood [32].

Functional TLRs activate the NF-kB pathway in several cell types, including neurons [33] and EGCs [21]. This transcription factor has been shown to convey signals from lipopeptides, LPS and class B CpG ODNs, leading to production of cytokines and chemokines [13, 14, 34]. Our results indicate that NF-kB is also involved in ENS-mediated recognition of MAMPs, but additionally suggest that other signalling cascades may participate in cytokine and chemokine production. Indeed, inhibition with Bay 11-7082 resulted in significant decrease in TNF-α, IL-6 and MCP-1 release, but not complete abrogation. Therefore, it is likely that other pathways, such as the MAPK and the 5-adenosine monophosphate-activated protein kinase, are responsible for the observed mediator production after NF-kB blockade, as they have been previously shown to play important roles in production of TNF-α in embryonic ENS culture [7] and central nervous system neurons [29].

Release of cytokines and other immunomodulatory molecules is a common feature following TLR recognition of MAMPs [13]. However, a number of adaptions have developed in the gastrointestinal tract to prevent TLR-driven immune responses to commensal microbes [35]. Some of these adaptions might also occur in the ENS, as we observed no significant pro-inflammatory or chemoattractive responses in embryonic ENS culture upon Pam2CSK4 or ODN 1826 stimulation, despite the fact that they induced NF-kB activation. Similar observations have been reported in human EGCs, in which translocation into the nucleus of the NF-kB p50 subunit takes place after exposure to enteropathogenic bacteria, but not to probiotic strains [21]. These findings might underlie selective mechanisms to signal microbe presence while minimising responses to those non-pathogenic.

We have reported an active participation of resident macrophages in IL-6 and MCP-1 production, which poses an important limitation to the use of these embryonic ENS cultures for immunologic research purposes. Indeed, the neuronal or macrophage origin of the released cytokines could not be quantified, making it difficult to define the actual contribution of each cell type to the inflammatory microenvironment and chemoattractive effects. We propose a major role for enteric neurons because (1) expression of TLR2/4/9 was preferentially seen in these cells; (2) TNF-α expression, which has been localised by our collaborators in enteric neurons following LPS exposure [7], was altered upon TLR2/4 and TLR4/9 interactions; (3) MCP-1 reactivity following LPS stimulation accumulated majorly in neurons and (4) TLR2 up-regulation upon LPS challenge occurred in neurons [7]. Even though the involvement of resident immunocytes may be important, embryonic macrophages display important phenotypic differences with adult macrophages, such as no major histocompatibility complex (MHC) class II expression or poor cytokine production [36, 37]. Similarly, embryonic EGCs did not release IL-6 upon MAMP challenge, which is in contrast with previous observations in adult cells [8, 21].

Consistent up-regulation of TLR2 following MAMP or cytokine stimulation has been described in astrocytes, neurons and other cell types [7, 24, 38, 39]. Induction of TLR2 mRNA is dependent on reactive oxygen species [39] and ultimately on NF-kB, which has different binding sites on the TLR2 promoter region [40]. Overexpression of this receptor is necessary to reach maximal NF-kB activation after MAMP recognition [38], possibly through signalling the formation of lipoproteins bearing lipid oxidation end-products [41, 42]. Our experiments using a TLR2-blocking antibody agree with these explanations, showing that in our setup, TLR2 induction is crucial to obtain maximal TNF-α, IL-6 and MCP-1 production following LPS stimulation. Furthermore, increased chemoattraction after combined challenge of TLR2/4 suggests that these receptors may interact in ways other than those we determined to enhance the release of cytokines or chemokines. These additional interactions might implicate activation of additional signalling pathways, since we report reduced responsiveness to NF-kB inhibitors in ENS-mediated release of IL-6 and MCP-1 after Pam2CSK4 + LPS stimulation.

In addition to TLR2/4 cross-responsiveness phenomena [43, 44], other TLR interactions after combined MAMP challenge have been documented [23, 45, 46]. Here, we demonstrate strong synergic responses in TNF-α release after costimulation with TLR4/9 ligands. Furthermore, IL-6 production and chemoattraction of macrophages by supernatants from ODN 1826 + LPS-treated embryonic cultures were also significantly enhanced, suggesting that additional chemokines might be induced after combined TLR4/9 challenge. Similar interactions between both receptors have been characterised in microglia, dendritic cells and bone marrow-derived macrophages [23, 46, 47]. The molecular mechanisms which may account for such effects are TLR9 up-regulation following LPS stimulation, as we and others have described [46], as well as enhanced signalling and duration through alternative pathways, such as the MAPK [47]. From a functional point of view, the ability to integrate different stimuli from the same microorganism contributes to discrimination of commensals and pathogens, inducing robust pro-inflammatory responses against the latter [48]. Recognition of Gram-negative motifs from both membrane (LPS) and nucleus (CpG DNA) is hence integrated in a synergic way, triggering strong pro-inflammatory responses and promoting chemoattraction of immune cells. In contrast, detection of Gram-positive and Gram-negative molecular patterns (Pam2CSK4 and LPS) lead to similar chemotactic responses but to a not so harsh pro-inflammatory environment, which might result into a milder priming of the subsequent effector response.

Different studies have addressed the involvement of central and peripheral nervous system neurons in defence against pathogens. These studies demonstrate that LPS stimulation activates NF-kB and MAPK pathways in these cells to induce production of cytokines like TNF-α and IL-6, and chemokines such as RANTES and KC [29, 33]. Participation of enteric neurons in inflammation has been also demonstrated. Transgenic mice displaying increased and reduced numbers of neurons develop, respectively, more and less severe experimental colitis than wild-type littermates, suggesting that neurons play pro-inflammatory roles [4]. Furthermore, different signalling pathways are activated in these cells upon IL-1β or LPS challenge [7, 49], leading to production of IL-8 and TNF-α [6, 7]. Our findings show that enteric neurons express TLR2/4/9,signal through the NF-kB pathway and release IL-6 and MCP-1 in response to LPS challenge. These observations add further evidence to the pro-inflammatory roles of neurons, and set them as active players in neuroimmune interactions in the gastrointestinal tract, participating in microbial recognition and priming subsequent immune responses.

## Conclusions

Our data suggest that the ENS is capable of recognising and discriminating between different microbial challenges, triggering robust or moderate inflammatory responses depending on the combination of TLR stimulated, as well as interacting with resident immunocytes to induce chemoattraction of macrophages. Given the fact that neurons are the main TLR-expressing cells in the enteric plexuses and are activated upon MAMP challenge to produce TNF-α, IL-6 and MCP-1, they arise as important players in enhancement of neuroimmune interactions.

## Abbreviations

DAPI, 4',6-diamidino-2-phenylindole; EGC, enteric glial cell; ENS, enteric nervous system; GFAP, glial fibrillary acidic protein; HRP, horseradish peroxidase; IBA-1, ionized calcium-binding adapter molecule; IkB, inhibitor of kB; IL-6, interleukin-6; LMMP, longitudinal muscle myenteric plexus; LPS, lipopolysaccharide; MAMP, microorganism-associated molecular pattern; MAPK, mitogen-activated protein kinase; MCP-1, monocyte chemoattractant protein-1; NF-kB, nuclear factor-kB; ODN, CpG oligonucleotide; PBS, phosphate-buffered saline; PCR, polymerase chain reaction; SMP, submucosal plexus; TLR, toll-like receptor; TNF-α, tumour necrosis factor-α

## Additional files

Additional file 1: TLR2/4/9 antibodies specifically stain ENS structures in WT mouse tissues. Tissue from WT, TLR2^−^^/^^−^ and TLR4^−^^/^^−^ mice was stained with TLR2/4 antibodies to test their staining specificity. In micrographs from WT mice, positive neurons were distinguished by strong immunoreactivity (white arrows) when compared to negative ones (open arrows). Knockout mice tissues displayed background immunoreactivity in neurons and unspecific secondary antibody precipitates. Pictures in this Add. File were taken in a Leica TCS SP5 microscope. Scale bars: 25 μm. The unconjugated TLR9 antibody (clone 26C593.2) raised in mouse could not be used to detect this receptor in mouse whole mounts, due to secondary antibody cross-reactivity with neurons, muscle fibres and supportive cells in the submucosa (TLR9 MOM). Conversely, the biotinylated form of this antibody displayed strong staining in the LMMP (black arrows) that was not observed in TLR9^−^^/^^−^ preparations (Biot TLR9). Antigen retrieval was achieved by boiling sections in 10 mM sodium citrate pH 6, followed by quenching of peroxidases and avidin/biotin blocking. After primary antibody incubation overnight at 4 °C, sections were washed, incubated with ABC-HRP kit and developed with DAB (both from Vector Laboratories). Haematoxylin was used to counterstain tissues. Scale bars: 20 μm. (TIF 12107 kb) Additional file 2: TLR2/4/9 display minor colocalisation with GFAP in adult rat plexuses. TLR colocalisation with the EGC marker GFAP in whole-mount preparations of the SMP and the LMMP. White arrows point to enteroglial processes colocalising with TLR expression. Scale bars: 25 μm. (TIF 2537 kb) Additional file 3: TLR2 colocalises with glial structures in adult LMMP. Different insets from LMMP micrographs in Fig. 1 and Additional file 2 were generated to demonstrate colocalisation between TLR2 and GFAP-expressing EGCs (arrows). (TIF 2932 kb) Additional file 4: Time-course activation of NF-kB and dose-dependence. Time-course activation after stimulation of ENS cultures with (A) 100 ng/mL Pam2CSK4, (B) 100 ng/mL LPS, (C) 1 μM ODN 1826 or (D) 1 μM control ODN 1826. (E) Quantification of the overall NF-kB activation after treatment with different doses of the indicated MAMPs. Densitometric measurements for each experiment were represented as time-course curves by means of the GraphPad Prism 5.0, and the area under the curve was calculated for each replicate. The resulting values were used to compare the NF-kB inducing strength of each TLR ligand (n = 4; **P* < 0.05, ***P* < 0.01 and ****P* < 0.001, one-way ANOVA followed by Dunnett’s post hoc test). Statistics were performed independently for each ligand. (TIF 13694 kb) Additional file 5: Resident immunocytes, but not EGCs, express IL-6. ENS cultures were costained for S100β (EGC marker), IBA-1 (macrophage marker) and IL-6 after 24 h stimulation with LPS. Scale bars: 25 μm. (TIF 1791 kb) Additional file 6: MCP-1 staining is more prominent in TLR4-expressing cells. ENS cultures were stimulated for 24 h with LPS and costained for MCP-1 and TLR4. Although cells displayed different staining intensities for TLR4, positive populations showed increased MCP-1 intensity (open arrows). MCP-1 was also observed in TLR4-negative cells, presumably macrophages, but the staining intensity was lower (white arrows). Scale bars: 25 μm. (TIF 857 kb) Additional file 7: Neurons are the only TLR4-expressing cells in ENS cultures. ENS cultures were stimulated for 24 h with LPS and costained for Hu C/D and TLR4. IL-6 antibody was also added to visualise macrophages (arrows). TLR4 was located exclusively in Hu C/D positive cells. Scale bars: 25 μm. (TIF 1806 kb)
