# Supplementary figures and images for: TLR2 and TLR9 modulate enteric nervous system inflammatory responses to lipopolysaccharide
Source: J Neuroinflammation. 2016 Aug 18;13:187. doi: 10.1186/s12974-016-0653-0 (PMC4990868; doi:10.1186/s12974-016-0653-0)

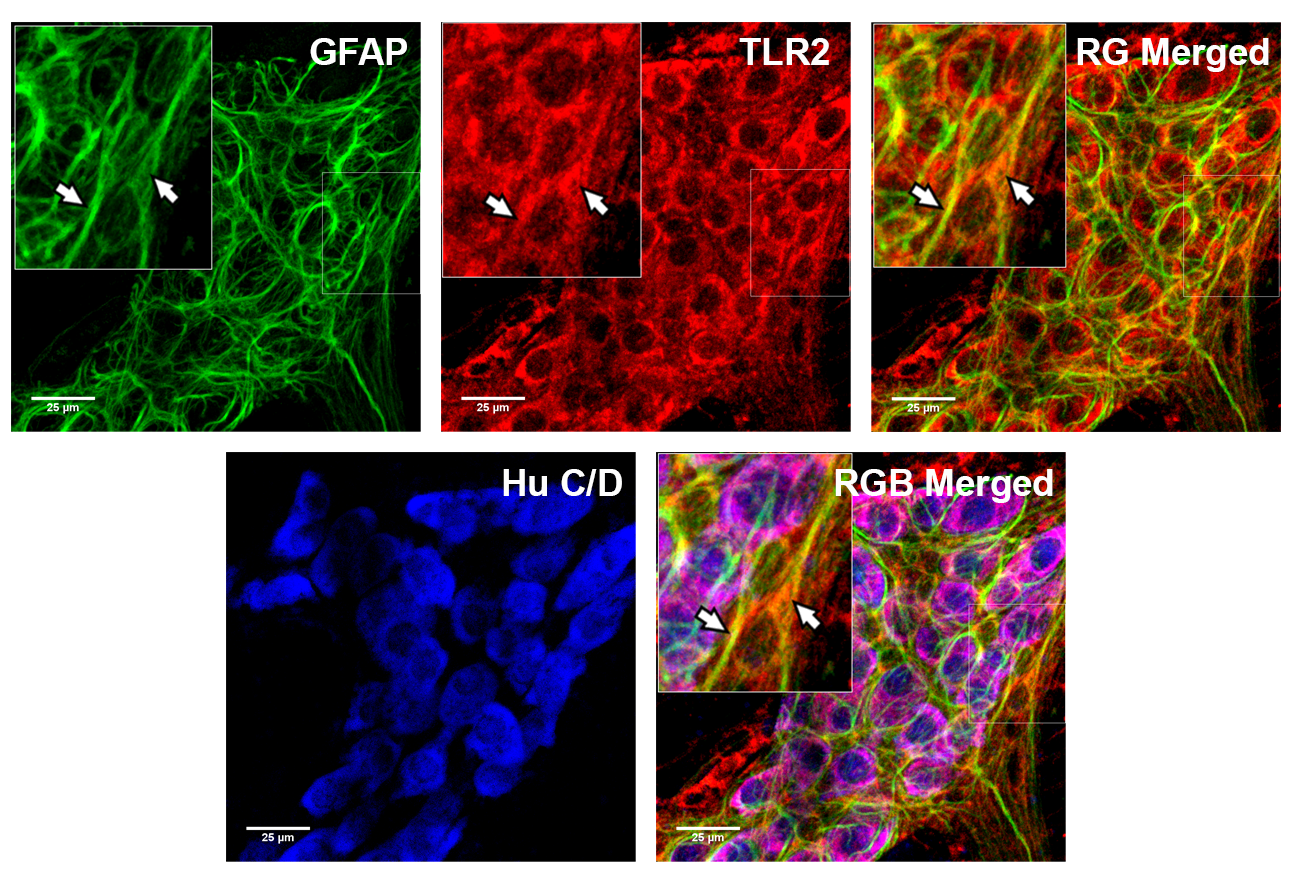

Supplement: Additional file 3: — TLR2 colocalises with glial structures in adult LMMP. Different insets from LMMP micrographs in Fig. 1 and Additional file 2 were generated to demonstrate colocalisation between TLR2 and GFAP-expressing EGCs (arrows). (TIF 2932 kb) [file 12974_2016_653_MOESM3_ESM.tif]

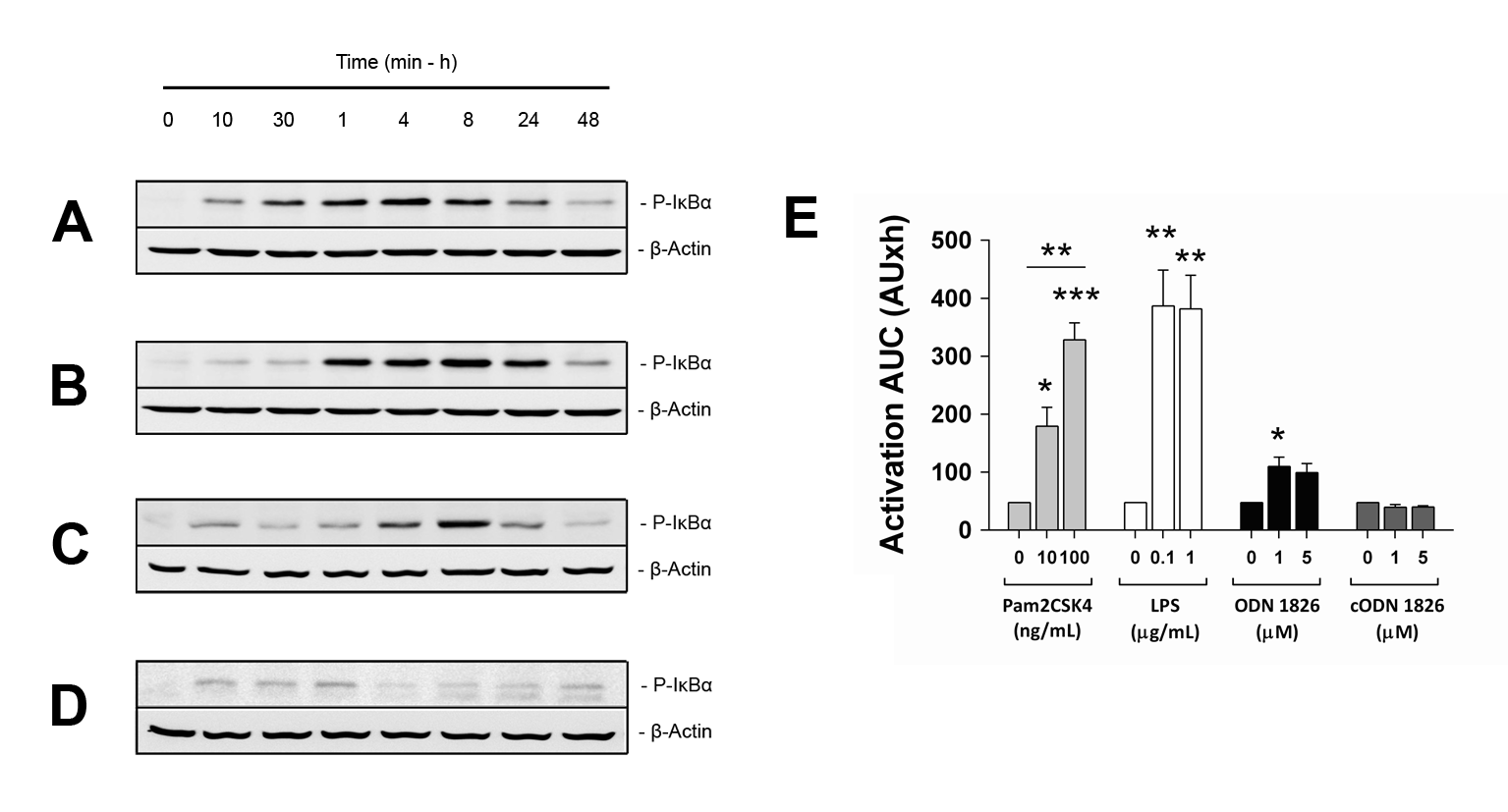

Supplement: Additional file 4: — Time-course activation of NF-kB and dose-dependence. Time-course activation after stimulation of ENS cultures with (A) 100 ng/mL Pam2CSK4, (B) 100 ng/mL LPS, (C) 1 μM ODN 1826 or (D) 1 μM control ODN 1826. (E) Quantification of the overall NF-kB activation after treatment with different doses of the indicated MAMPs. Densitometric measurements for each experiment were represented as time-course curves by means of the GraphPad Prism 5.0, and the area under the curve was calculated for each replicate. The resulting values were used to compare the NF-kB inducing strength of each TLR ligand (n = 4; *P < 0.05, **P < 0.01 and ***P < 0.001, one-way ANOVA followed by Dunnett’s post hoc test). Statistics were performed independently for each ligand. (TIF 13694 kb) [file 12974_2016_653_MOESM4_ESM.tif]

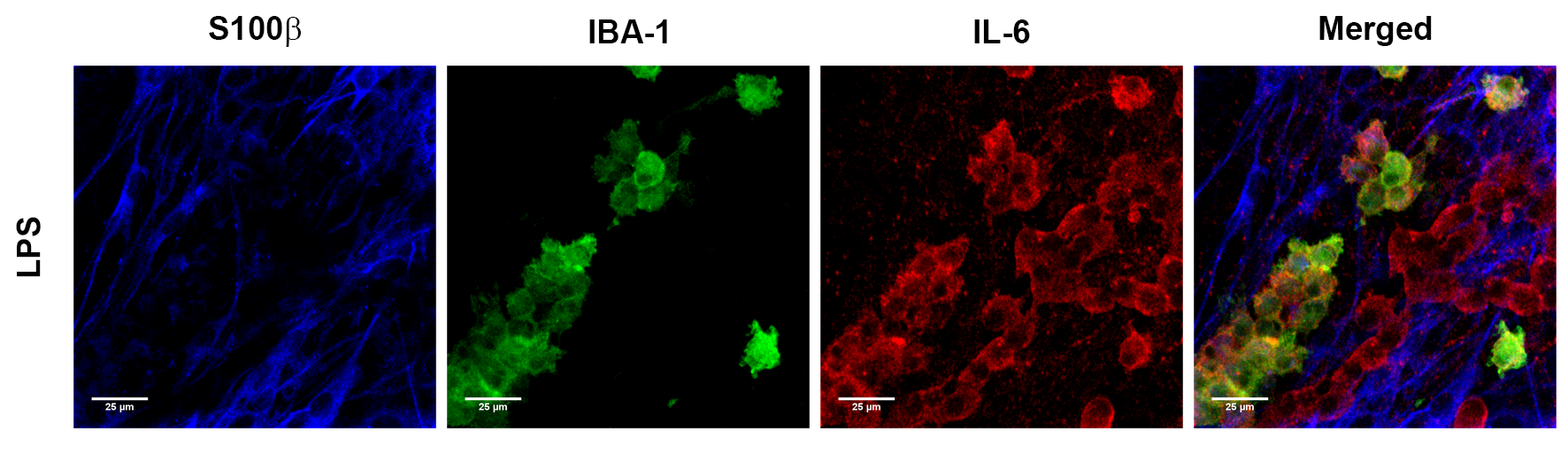

Supplement: Additional file 5: — Resident immunocytes, but not EGCs, express IL-6. ENS cultures were costained for S100β (EGC marker), IBA-1 (macrophage marker) and IL-6 after 24 h stimulation with LPS. Scale bars: 25 μm. (TIF 1791 kb) [file 12974_2016_653_MOESM5_ESM.tif]

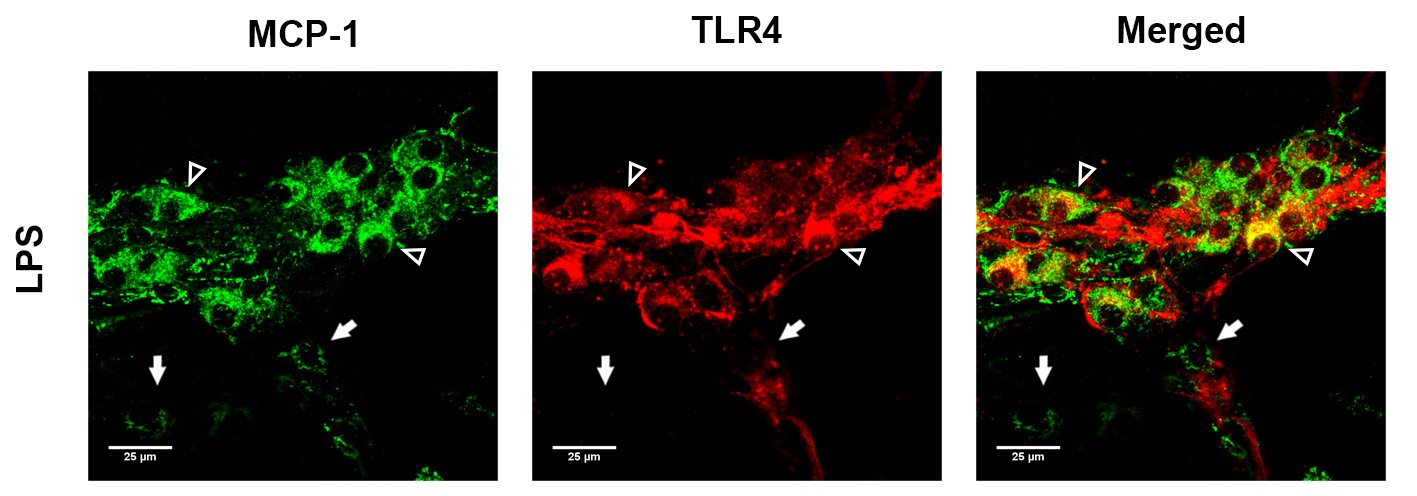

Supplement: Additional file 6: — MCP-1 staining is more prominent in TLR4-expressing cells. ENS cultures were stimulated for 24 h with LPS and costained for MCP-1 and TLR4. Although cells displayed different staining intensities for TLR4, positive populations showed increased MCP-1 intensity (open arrows). MCP-1 was also observed in TLR4-negative cells, presumably macrophages, but the staining intensity was lower (white arrows). Scale bars: 25 μm. (TIF 857 kb) [file 12974_2016_653_MOESM7_ESM.tif]

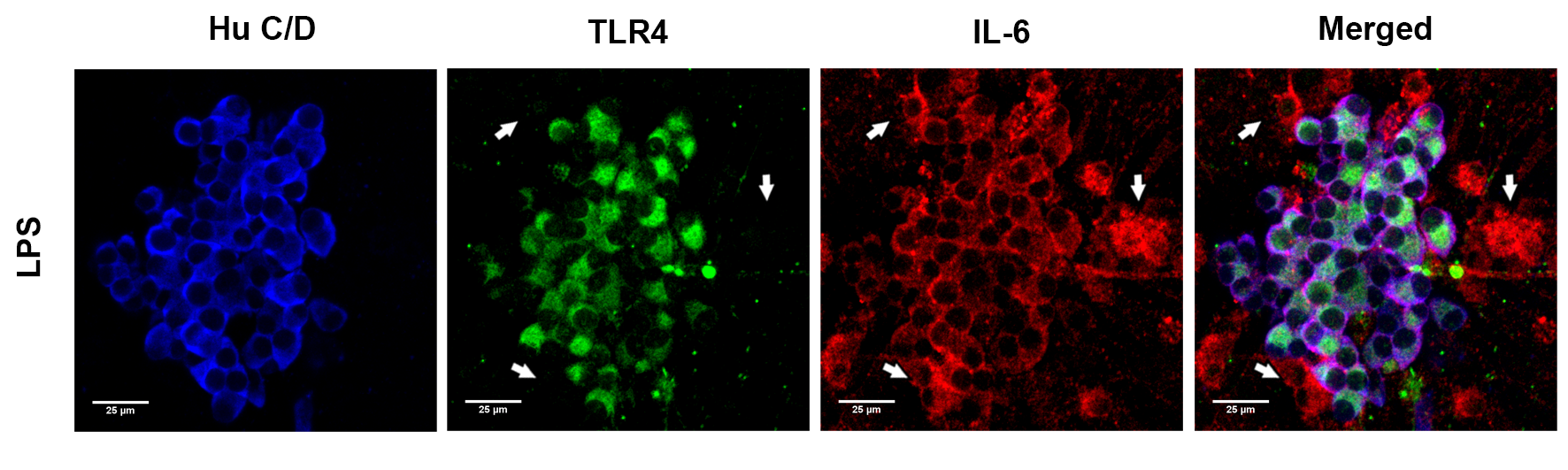

Supplement: Additional file 7: — Neurons are the only TLR4-expressing cells in ENS cultures. ENS cultures were stimulated for 24 h with LPS and costained for Hu C/D and TLR4. IL-6 antibody was also added to visualise macrophages (arrows). TLR4 was located exclusively in Hu C/D positive cells. Scale bars: 25 μm. (TIF 1806 kb) [file 12974_2016_653_MOESM6_ESM.tif]
